# Identification of Chromosome Segment Substitution Lines of *Gossypium barbadense* Introgressed in *G*. *hirsutum* and Quantitative Trait Locus Mapping for Fiber Quality and Yield Traits

**DOI:** 10.1371/journal.pone.0159101

**Published:** 2016-09-07

**Authors:** Huanchen Zhai, Wankui Gong, Yunna Tan, Aiying Liu, Weiwu Song, Junwen Li, Zhuying Deng, Linglei Kong, Juwu Gong, Haihong Shang, Tingting Chen, Qun Ge, Yuzhen Shi, Youlu Yuan

**Affiliations:** 1 State Key Laboratory of Cotton Biology, Key Laboratory of Biological and Genetic Breeding of Cotton, The Ministry of Agriculture, Institute of Cotton Research, Chinese Academy of Agricultural Sciences. Anyang, Henan, China; 2 College of Bioengineering, Henan University of technology, Zhengzhou, Henan, China; USDA-ARS Southern Regional Research Center, UNITED STATES

## Abstract

Chromosome segment substitution lines MBI9804, MBI9855, MBI9752, and MBI9134, which were obtained by advanced backcrossing and continuously inbreeding from an interspecific cross between CCRI36, a cultivar of upland cotton (*Gossypium hirsutum*) as the recurrent parent, and Hai1, a cultivar of sea island cotton (*G*. *barbadense*) as the donor parent, were used to construct a multiple parent population of (MBI9804×MBI9855)×(MBI9752×MBI9134). The segregating generations of double-crossed F_1_ and F_2_ and F_2:3_ were used to map the quantitative trait locus (QTL) for fiber quality and yield-related traits. The recovery rate of the recurrent parent CCRI36 in the four parental lines was from 94.3%–96.9%. Each of the parental lines harbored 12–20 introgressed segments from Hai1across 21 chromosomes. The number of introgressed segments ranged from 1 to 27 for the individuals in the three generations, mostly from 9 to 18, which represented a genetic length of between 126 cM and 246 cM. A total of 24 QTLs controlling fiber quality and 11 QTLs controlling yield traits were detected using the three segregating generations. These QTLs were distributed across 11 chromosomes and could collectively explain 1.78%–20.27% of the observed phenotypic variations. Sixteen QTLs were consistently detected in two or more generations, four of them were for fiber yield traits and 12 were for fiber quality traits. One introgressed segment could significantly reduce both lint percentage and fiber micronaire. This study provides useful information for gene cloning and marker-assisted breeding for excellent fiber quality.

## Introduction

Cotton is one of the most important cash crops in the world and cotton fiber provides the main natural raw material for the textile industry. Upland cotton (*Gossypium hirsutum*) has a high yield and wide adaptability, but a relatively low fiber quality. On the other hand, sea island cotton (*G*. *barbadense*) has excellent fiber quality with low yield and limited adaptability. Therefore, one way to improve fiber quality of upland cotton is to introgress the favorable genes from sea island cotton to upland cotton. However, the yield and quality of cotton are quantitative traits that are affected by multiple genes and often negatively correlated [1–3]. Therefore, the simultaneous improvement of cotton fiber quality and yield is a tall task for breeders in conventional breeding [4]. With the continuous development and improvement of molecular marker technologies, researchers have conducted extensive studies to construct cotton genetic maps and identify quantitative trait locus (QTL). This would make it possible to simultaneously improve both of fiber quality and yield in a breeding program.

The construction of the first cotton molecular genetic map [5] had facilitated QTL mapping. Completion of the cotton genome draft sequence laid a foundation for further molecular design breeding at the whole genomic level [6–9]. Most segregating populations for QTL identification are F_2_ [10–12], BC_1_ [13], BIL [14] and RIL [15–18]. However, because these segregating populations (e.g., F_2_) are usually not immortal, the results of QTL identification are usually difficult to repeat. Furthermore, although several QTLs for cotton fiber quality or yield traits have been identified, fine mapping and cloning of these genes have rarely begun. Chromosome segment substitution lines (CSSLs), also known as introgression lines, are permanent populations that possess the same genetic background as the recurrent parent. Differences among CSSLs usually involve only one or a few of the introgressed chromosome segments, which in turn effectively eliminate interference of the genetic background. CSSLs are also highly efficient in detecting QTLs with minor effects. Therefore, CSSLs are ideal materials for QTL fine mapping, gene cloning, and investigating QTL interactions. Since Eshed and Zamir first constructed introgression lines of tomato [19], these have been successfully applied in rice, corn, and other plants [20–23].

CSSLs are seldom reported in QTL studies in cotton. Stelly et al. first constructed 17 chromosome substitution lines of *G*. *barbadense* in TM-1 background of *G*. *hirsutum* [24]. Subsequently, the same research team performed a thorough analysis of the genetic effects of CSSLs [25–31]. Their results showed that the sea island cotton genotype has positive effects on fiber quality traits, suggesting that these particular traits are influenced by multiple genes [32]. Other researchers also used CSSLs for QTL mapping of fiber quality and yield traits [33–37]. Although these CSSL populations are beneficial for QTL mapping, a large gap between the QTL mapping and the application of these lines in breeding programs remains to be resolved.

To simultaneously obtain brand-new lines for direct application in breeding while conducting basic researches, we constructed a CSSL population using upland cotton CCRI36 and sea island cotton Hai1, both of which are commercially grown cultivars. A genetic linkage map containing 2,292 markers was constructed [38] and cotton fiber quality and yield-related QTLs were identified in this CSSL population. Liang et al. [39] detected 20 yield-related QTLs in BC_5_F_2_ of this CSSL population. Through multi-ecological environment evaluations of the yield and fiber quality of the population (BC_5_F_3_, BC_5_F_3:4_, and BC_5_F_3:5_), Zhang et al. [40] and He et al. [41] mapped specific QTLs for fiber quality and yield-related traits using selected lines with stable and excellent fiber quality and yield.

In the present study, based on the phenotypic performance of this CSSL population [38], as well as previous findings from multi-ecological environment investigations [38–41], four introgression lines MBI9804, MBI9855, MBI9752, and MBI9134 with excellent fiber quality were selected as parental lines, and a double-crossed population of (MBI9804×MBI9855)×(MBI9752×MBI9134) was constructed. The introgressed Hai1 segments were evaluated in the segregating generations F_1_ and F_2_ and verified in the following F_2:3_ generation using SSR markers. We also performed QTL mapping for fiber quality and yield related traits with these three generations. New stable QTLs for fiber quality and yield traits were identified and validated in multiple generations. Our study lays a foundation for fine mapping of fiber quality QTLs and using them in breeding via marker assisted selection (MAS).

## Materials and Methods

### Materials

CSSL population was constructed by crossing and backcrossing between donor parent Hai1 and the recurrent parent CCRI36. Hai1 was a commercially grown cultivar of *G*. *barbadense* with a dominant glandless gene, highly resistant to *Verticillium* wilt, and has excellent fiber quality [42]. While CCRI36 was a widely grown upland cotton cultivar with high yield and early maturity, and developed by the Institute of Cotton Research of Chinese Academy of Agricultural Sciences (State Approval Certificate of Cotton 990007). The development of the CSSL population was reported by Shi et al. [38]. The four BC_5_F_4_ introgression lines, MBI9804, MBI9855, MBI9752, and MBI9134, with stable and excellent fiber quality performance, were selected as parental lines to construct a double-crossed population of (MBI9804×MBI9855) × (MBI9752×MBI9134). In 2012, the double-crossed F_1_ was planted in the experimental farm (Anyang, Henan Province) of the Institute of Cotton Research of Chinese Academy of Agricultural Sciences. All parental lines were planted in two rows and a total of 868 individuals of double-crossed F_1_ were planted in 45 rows. Each row was 5m long and 0.8m apart with 20 plants. The F_1_ plants were selfed and their fiber and seeds were harvested by individual. In 2013, A total of 839 F_2_ individual plants were randomly selected from mixture seeds of all F_1_ plants in Anyang in 45 rows, with row length of 5 m, row spacing of 0.8 m, and plant spacing of 0.25 m. In 2014, 237 F_2:3_ families were randomly selected with the different fiber quality and planted in Shihezi, Xinjiang Autonomous Region, in two-narrow-row plots, with row length of 3 m and plant spacing of 0.12 m. The plastic-membrane covering technique and a wide/narrow row spacing pattern were used. Row spacing alternation was 0.2 m and 0.6 m.

### Investigation of Fiber Yield and Quality Traits

In 2012 and 2013, the phenotypic traits of each plant were investigated. Naturally opened bolls per plant were harvested for indoor testing, including seed cotton weight, fiber weight, boll weight (BW), lint percentage (LP), fiber length (FL), fiber uniformity (FU), fiber micronaire (FM), and fiber strength (FS). In 2014, 30 naturally opened bolls for each plot were harvested for indoor testing same as those in 2012 and 2013. The fiber quality traits were tested with HFT9000 in the Cotton Quality Supervision and Testing Center of the Ministry of Agriculture of China. HVICC international calibration cotton samples were used.

### DNA Extraction and SSR Molecular Detection

In 2012 and 2013, young leaves of the parental lines and the double-crossed F_1_ and F_2_ individual plants were sampled. DNA was extracted using a modified CTAB method [43]. SSR amplification and polyacrylamide gel electrophoresis were performed following Zhang’s description [44]. Based on the previously constructed CCRI36×Hai1 BC_1_F_1_ genetic linkage map [38], SSR markers were selected at a distance of 10–20 cM. A total of 526 pairs of polymorphic SSR markers between CCRI36 and Hai1 were selected for screening of polymorphisms among the four parental lines. Finally, 51 out of the 526 markers were identified to be polymorphic for genotyping of the double-crossed F_1_ and F_2_ individual plants. The sequences of the SSR primers were uploaded to the CMD database (http://www.cottonmarker.org/). The primers used in the present study were synthesized by Beijing Sunbiotech Co., Ltd. (Beijing, China).

### Analysis of Phenotypic Traits

EXCEL 2013 software was used for the descriptive statistical analysis of fiber quality traits (including FL, FS, FM, and FU) and yield related traits (including BW and LP) for the double-crossed F_1_ and F_2_ individual plants and the F_2:3_ family lines. The statistical values included average, maximum, minimum, the transgressive rate over the recurrent parent (%), coefficient of variation, skewness and kurtosis. Correlation analysis and ANOVA were performed using the SPSS20.0 software.

### Genotypic Analysis for Parents and Population

Genotypic analysis of the parental lines and population was performed based on the SSR polymorphic results using the GGT2.0 software developed by van Berloo (http://www.plantbreeding.wur.nl/UK/software_ggt.html) [45]. The background recovery rate of the CSSLs to the recurrent parent, number of introgressed segments, and length were calculated.

### QTL mapping

The linkage map was constructed using the MapChart2.2 software [46]. QTL mapping was performed using the QTL IciMappingV4.0 software developed by Wang et al. [47]. The nomenclature of QTL was: q + trait abbreviation + chromosome number + serial number of the marker closely linked to the trait. For example, qFS-2-7 represented a QTL controlling fiber strength near the seventh marker on chromosome 2 (Chr2).

## Results

### Fiber Quality and Yield Traits and Correlation Analysis

The four parents had longer fiber than the recurrent parent CCRI36 (P < 0.05). MBI9804 and MBI9134 had significant stronger fiber than that of CCRI36 (P < 0.01) (Tables 1 and 2). In the three generations, except for FU in 2013 and FM in 2014, the average values of the other traits were higher than those of the recurrent parent CCRI36 (P < 0.05). The absolute value of the skewness was <1, indicating that the fiber quality and yield traits showed a normal distribution in the three generations. The recovery rates to the recurrent parent CCRI36 for FL, FU, FM, and FS increased from F_1_ to F_2:3_, whereas those of BW and LP decreased. The coefficient of variation indicated that fiber quality traits, FM, FS, and FL were highly variable compared to FU.

**Table 1:** Phenotypic analysis of parent fiber quality and yield traits.

Parents	Environment	FL(mm)		FU(%)		FM(unit)		FS(cN/tex)		BW(g/boll)		LP(%)	
		Mean	SD	Mean	SD	Mean	SD	Mean	SD	Mean	SD	Mean	SD
CRI36	2012AY	29.14	1.48	85.18	1.56	4.25	0.45	29.53	1.8	5.05	0.71	36.45	4.73
	2013AY	28.29	0.82	85.2	0.9	4.51	0.11	29.9	1.65	5.4	0.63	37.86	1.77
	2014XJ	28.69	0.6	85.25	1.1	4.27	0.38	29.8	1.46	5.17	0.44	40.22	0.01
MBI9804	2012AY	30.83**	1.63	85.90**	1.55	4.10**	0.47	30.45**	1.15	4.8	0.87	34.45	3
	2013AY	29.93*	1.2	85.99	1.17	4.59	0.12	32.79**	1.58	4.95	0.47	32.87	1.24
MBI9855	2012AY	32.22**	1.61	85.6	1.19	3.6	0.45	32.65	1.28	4.80*	0.78	34.50*	4.08
	2013AY	30.99**	0.81	85.64	0.65	3.71**	0.08	33.12	1.47	5.50*	0.35	30.82*	1.44
MBI9752	2012AY	29.34**	1.03	83.9	1.48	4.01	0.43	31.2	1.32	5.05	0.64	30.1	3.51
	2013 AY	29.32*	1.34	86.15	0.95	4.38	0.12	31.65	1.64	4.8	0.53	35.52	2.83
MBI9134	2012AY	30.22**	2.05	84.75**	1.45	4.02**	0.35	30.40**	1.62	4.02	0.68	34.15	6.18
	2013AY	29.84*	1.01	84.85	0.99	4.17	0.17	32.06**	1.75	4.47	0.47	34.3	4.17

Note: AY: Anyang; XJ: Xinjiang

** indicates P < 0.01;

* indicates P < 0.05

**Table 2:** Phenotypic analysis of fiber quality and yield traits in the three populations.

Trait	Year	CCRI36				generation								
		Mean	Min.	Max.	SD		Mean	Min.	Max.	SD	Transgressive rate (%)	CV(%)	Skewness	Kurtosis
FL (mm)	2012	29.14	24.65	30.28	1.48	F_1_	29.39**	23.88	33.63	1.49	86.29	5.06	-0.45	0.11
	2013	28.29	27.41	30.14	0.82	F_2_	30.26**	25.67	34.00	1.23	85.94	4.06	-0.01	0.08
	2014	28.69	27.51	29.77	0.60	F_2:3_	30.43**	27.28	34.08	1.21	94.09	3.99	0.27	0.19
FU(%)	2012	85.18	81.40	86.20	1.56	F_1_	84.48**	88.00	88.00	1.48	72.58	1.76	-0.49	0.18
	2013	85.20	83.50	86.50	0.90	F_2_	85.82	89.20	89.20	1.28	73.30	1.49	-0.73	1.63
	2014	85.25	85.25	83.50	1.10	F_2:3_	86.06*	89.20	89.20	0.97	73.84	1.13	-0.21	0.34
FM	2012	4.25	2.61	4.33	0.45	F_1_	3.65	2.06	7.18	0.58	39.29	16.03	0.05	3.90
	2013	4.51	3.88	4.92	0.11	F_2_	4.07**	2.71	5.44	0.46	80.70	11.30	-0.29	0.02
	2014	4.27	3.69	4.91	0.38	F_2:3_	4.00**	2.93	5.09	0.38	84.81	9.44	0.00	-0.16
FS(cN/tex)	2012	29.53	25.20	41.40	1.80	F_1_	29.58**	24.30	34.40	1.71	72.35	5.78	-0.29	-0.27
	2013	29.90	29.00	34.80	1.65	F_2_	32.65**	26.40	41.50	2.21	78.55	6.77	0.21	0.15
	2014	29.80	27.40	32.70	1.46	F_2:3_	32.68**	26.90	37.50	1.80	90.72	5.52	-0.09	0.42
BW	2012	5.05	2.85	5.20	0.71	F_1_	4.57*	2.57	7.96	0.73	57.83	15.86	0.24	0.24
	2013	5.40	4.09	6.36	0.63	F_2_	4.90**	2.69	8.42	0.70	46.18	14.36	0.39	1.30
	2014	5.17	4.67	6.65	0.44	F_2:3_	5.16*	3.48	6.24	0.38	41.07	7.44	-0.34	1.25
LP(%)	2012	36.45	19.75	37.24	4.73	F_1_	31.26**	14.22	50.76	0.03	94.70	8.96	-0.16	3.87
	2013	37.86	34.76	39.31	1.77	F_2_	32.38	22.58	57.15	0.03	36.47	10.45	0.38	3.09
	2014	40.22	36.47	41.18	0.01	F_2:3_	36.79**	27.72	45.66	0.03	18.07	7.07	-0.22	0.82

Note:

** indicates P < 0.01;

* indicates P < 0.05

Correlation analysis between fiber quality and yield traits (Table 3) showed that FS was significantly positively correlated with FL in all three generations, significantly positively correlated with FU in F_1_ and F_2_. FL was significantly positively correlated with FU in F_1_ and F_2_, significantly negatively correlated with FM in F_2_ and F_2:3_, whereas significantly positively correlated were FM in F_1_.

**Table 3:** Correlation between fiber quality and yield traits in the three populations.

traits	Population	FL	FU	FM	FS	LP
FU	F_1_	0.465**				
	F_2_	0.274**				
	F_2:3_	0.037				
FM	F_1_	0.106**	0.402**			
	F_2_	-0.203**	0.239**			
	F_2:3_	-0.370**	0.093			
FS	F_1_	0.566**	0.528**	-0.022		
	F_2_	0.504**	0.290**	-0.008		
	F_2:3_	0.724**	0.117	-0.312**		
LP	F_1_	0.133**	0.155**	0.101**	0.004	
	F_2_	-0.108**	-0.018	0.375**	-0.257**	
	F_2:3_	-0.213**	-0.004	0.533**	-0.252**	
BW	F_1_	0.327**	0.460**	0.452**	0.320**	-0.055
	F_2_	0.054	0.155**	0.250**	0.125**	-0.133**
	F_2:3_	-0.049	0.006	0.123	-0.031	-0.131*

Note:

** indicates *P* < 0.01 (two-sided);

* indicates *P* < 0.05 (two-sided).

### Genotypic Analysis of the Parents and Populations

**Fig 1:**
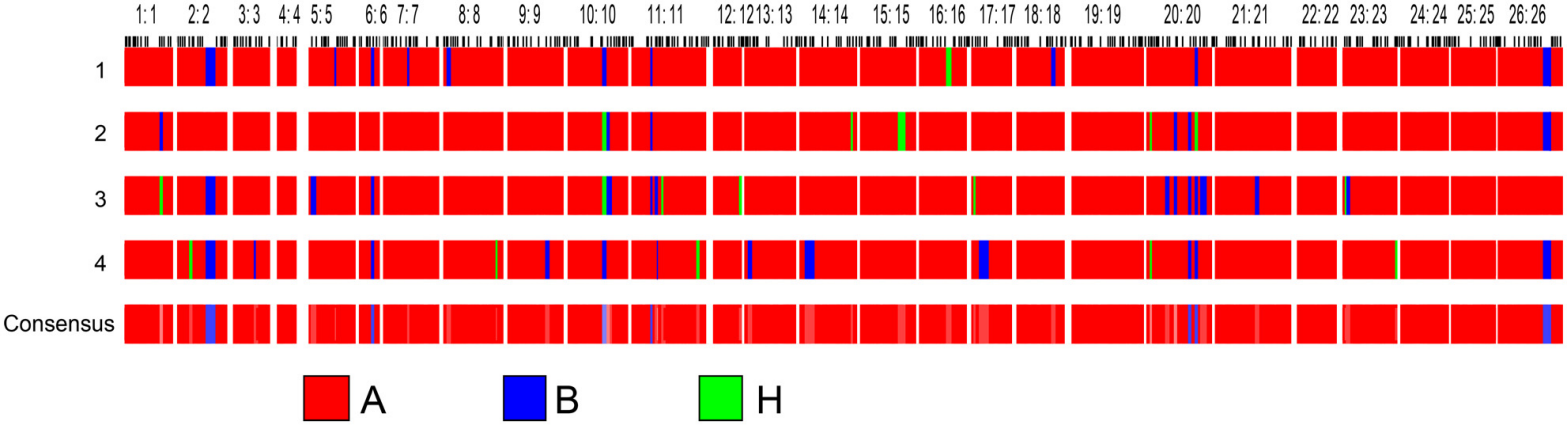
Introgressed Hai1 chromosome segments in the parents. 1: MBI9804; 2: MBI9855; 3: MBI9752; and 4: MBI9134; A: genome of recurrent parent CCRI36; B: homologous Hai1 segments; H: heterozygous Hai1segments.

Introgressed Hai1 chromosome segments in the four parental lines were identified by SSR markers using the GGT2.0 software (S1 Table, Fig 1). The background recovery rate in the four parental lines ranged from 94.1% to 97.6%. Each of the parental line harbored 12–20 introgressed Hai1 chromosome segments, spanning a genetic length of 116.3 cM–283.1 cM, and accounting for 2.4%–5.9% of the total detected genetic length. The introgressed Hai1 segments were distributed across 21 chromosomes in all four parental lines, mainly on Chr10, Chr11, Chr20 and Chr23. Chr20 had the most introgressed segment number, whereas no introgressed segments were detected on Chr4, Chr19, Chr22, Chr24 and Chr25. The number of homozygous introgressed Hai1 segments were more than that of the heterozygous introgressed segments in three parental lines, except for MBI9855.

Genotyping analysis (S2 Table) showed that the average recovery rates to CCRI36 in F_1_, F_2_ and F_2:3_ generations were 96.1%, 96.3% and 96.0%, respectively. The average length of the homozygous introgressed Hai1 segments ranged from 71.8–110.0 cM in the three generations, whereas the average length of the heterozygous introgressed Hai1 segments was 68.5–103.5 cM. The average length of the total introgressed Hai1 segments was 172.0–182.8 cM.

**Fig 2:**
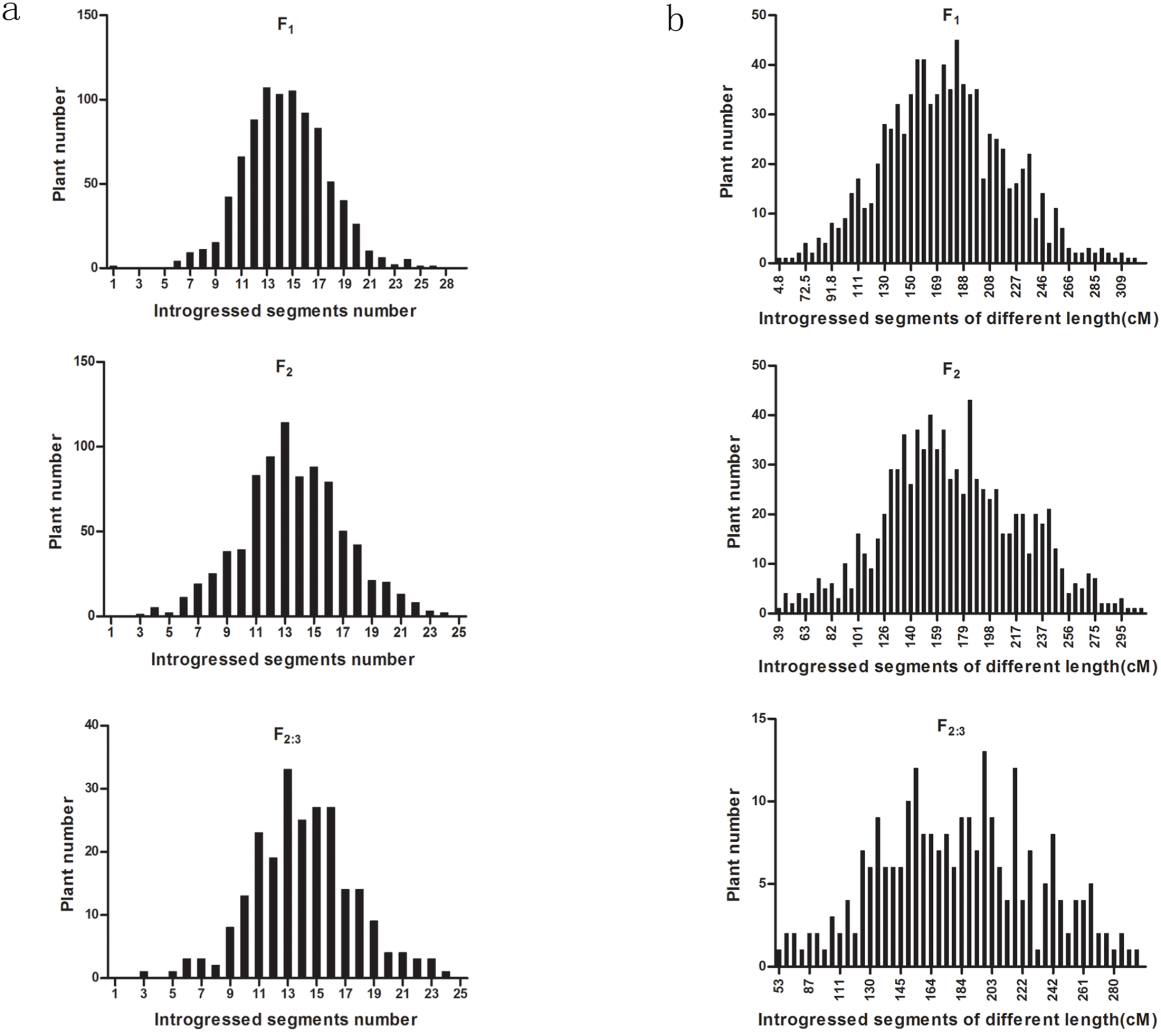
Numbers and total length of the introgressed Hai1 segments in the three generations. a: Number of the introgressed Hai1 segments; b: Total length of the introgressed Hai1 segments.

In all three generations, most individual plants contained 4 or more introgressed Hai1 segments, whereas a few plants contained 1–3 introgressed Hai1 segments. The minimum number of introgressed Hai1 segments was one and the maximum was 27, with an average of 13.6–14.5 introgressed Hai1 segments. The length of the introgressed segments was mainly between 126 cM and 246 cM. In F_2_, no heterozygous segments were detected in three plants. In F_1_, no homozygous segments were detected in four plants and one introgressed segment was detected in only one plant (S3 Table, Fig 2a and 2b).

### QTL Mapping

**Fig 3:**
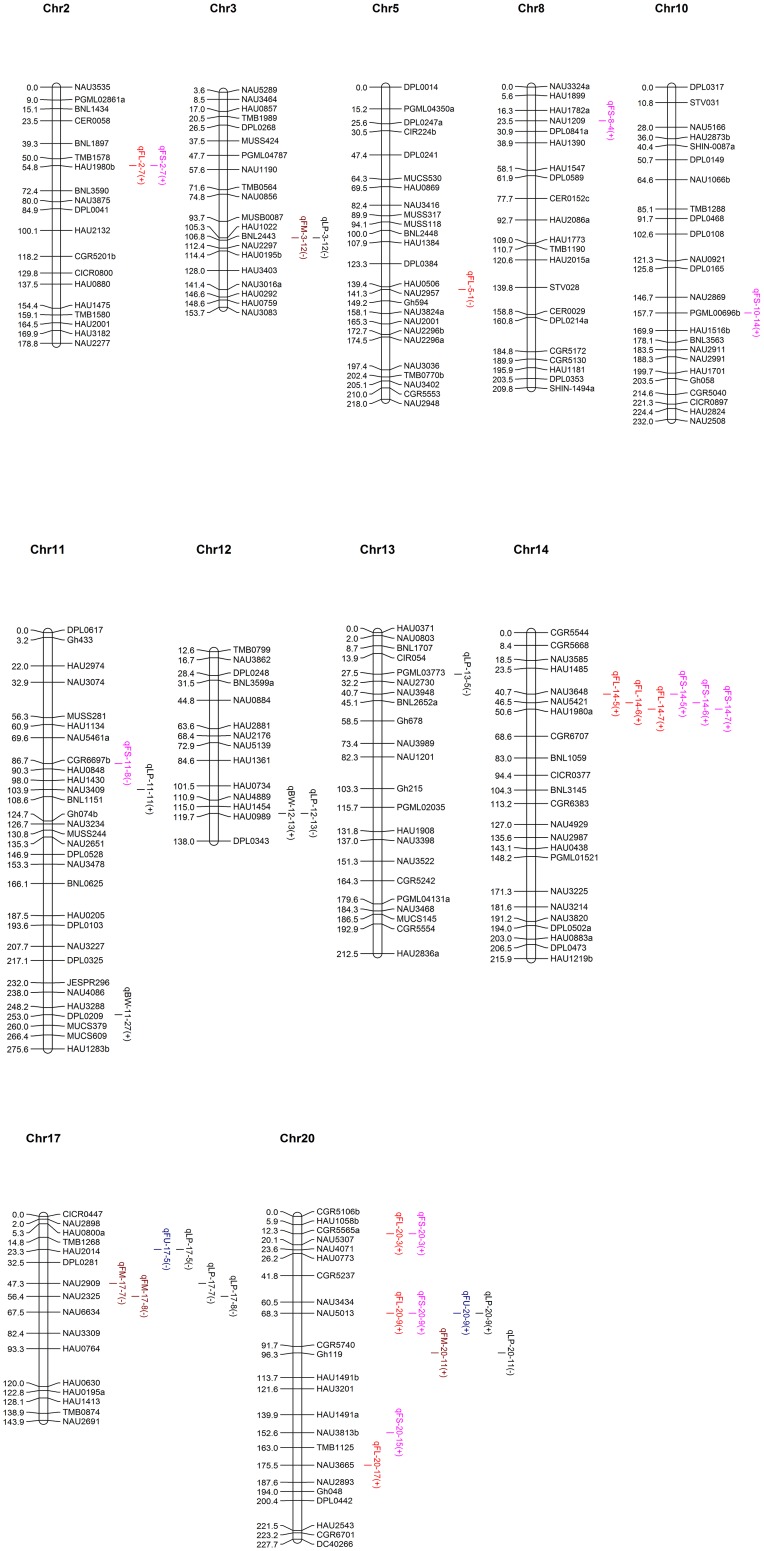
Mapping of QTLs for cotton fiber quality and yield traits on the linkage map.

A total of 35 QTLs for the yield and fiber quality traits was identified in F_1_, F_2_, and F_2:3_, including 24 QTLs controlling four cotton fiber quality traits and 11 QTLs controlling two cotton yield traits (S4 Table, Fig 3). These QTLs were distributed across 11 chromosomes, with LOD values between 2.31 and 26.38, collectively explained 1.78%–20.27% of the observed phenotypic variations. Three QTLs were detected in 3 generations, and the 13 QTLs were detected in two generations, these 16 QTLs were regarded as stable ones, of which included 12 fiber quality-related QTLs and 4 yield-related QTLs.

Fiber length: Eight QTLs controlling FL were detected on four chromosomes (Chr2, Chr14, Chr5 and Chr20), explaining 1.93%–19.68% of the observed phenotypic variations. Among these QTLs, one on Chr2 (qFL-2–7) and three on Chr14 (qFL-14-5, qFL-14-6 and qFL-14-7) were detected in both F_1_ and F_2_ generations. Three QTLs in F_2:3_ were mapped on Chr5 (qFL-5-1) and Chr20 (qFL-20-3 and qFL-20-17), explaining 4.91%, 5.37%, and 19.68% of the observed phenotypic variations, respectively. The negative additive effect for qFL-5-1 indicated that CCRI36 alleles increased fiber length.

Fiber strength: A total of 10 QTLs for FS were detected on six chromosomes (Chr2, Chr8, Chr10, Chr11, Chr14 and Chr20), explaining 1.84%–6.49% of the observed phenotypic variations. Among these QTLs, four that were detected in both F_1_ and F_2_ were mapped on Chr2 (qFS-2-7) and Chr14 (qFS-14-5, qFS-14-6 and qFS-14-7), explaining 2.07%–5.77%, 5.53%–6.49%, 5.06%–6.06%, and 5.24%–5.78% of the observed phenotypic variations, respectively. One QTL mapped on Chr20 was detected in F_2:3_ (qFS-20-3), explaining 4.73% of the observed phenotypic variations. Of the remaining QTLs, one was detected in F_1_ and four in F_2_.

Micronaire: Four QTLs for FM were mapped on three chromosomes (Chr3, Chr17 and Chr20), explaining 1.95%–11.69% of the observed phenotypic variations. Among these QTLs, three were detected both in F_2_ and F_2:3_, and mapped on Chr3 (qFM-3-12) and Chr17 (qFM-17-7 and qFM-17-8), explaining 8.86%–10.46%, 5.12%–11.69% and 6.78%–7.67% of the observed phenotypic variations, respectively. The negative additive effects indicated that CCRI36 alleles increased micronaire value.

Fiber uniformity: Two QTLs for fiber uniformity were mapped on Chr17 and Chr20. qFU-17-5 explained 2.49%–3.83% of the observed phenotypic variations with a negative additive effect. QTL qFU-20-9 was detected in both F_1_ and F_2_, explaining 3.57% of the observed phenotypic variations, with a positive additive effect which indicated Hai1 alleles increased fiber uniformity.

Boll weight: Two QTLs for BW were mapped on Chr11 and Chr12, explaining 6.02%–9.50% of the observed phenotypic variations. The positive additive effects indicated that Hai1 alleles increased boll weight.

Lint percentage: Nine QTLs for LP were mapped on 6 chromosomes (Chr3, Chr11, Chr12, Chr13, Chr17 and Chr20), explaining 1.84%–13.50% of the observed phenotypic variations. Three QTLs, qLP-3-12, qLP-17-7 and qLP-17-8, were detected in all F_1_, F_2_ and F_2:3_, explaining 8.01%–9.91%, 5.28%–13.5% and 4.4%–11.57% of the observed phenotypic variations, respectively. qLP-12-13 was detected in both F_2_ and F_2:3_, and explained 5.05%–10.77% of the observed phenotypic variations. The negative additive effects indicated that CCRI36 alleles increased lint percentage.

## Discussion

### Assessment and Application of Chromosome Segment Substitution Lines

CSSLs are usually applied to investigate the genetic behavior and effects of chromosome introgression **Segment** from the donor parent in the background of the recurrent parent. Its application for QTL mapping generally improves accuracy. CSSLs also provide permanent segregation populations for studying multi-environmental stability of the mapped QTLs. In the previous studies, a set of CSSL population was constructed using a widely planted upland cotton cultivar, CCRI36 as the recurrent parent, which is characterized of high yield and early maturity, and a sea island cotton Hai1 as the donor parent, which had good fiber quality and a high level of resistance to *Verticillium* wilt [38, 39, 40, 41]. In the current study, four introgression lines with excellent fiber quality which derived from this CSSL population were used to construct the segregating populations of double-crossed F_1_, F_2_, and F_2:3_. The introgressed Hai1 Chromosome segments were detected in individual plants of all three generations. The genotyping of each individual plant in the three generations was relatively clear. The recovery rate to the recurrent parent in all three generations was >95%. These introgression lines are ideal materials for further fine mapping, gene interaction analysis, heterosis mechanism study, and functional genomics.

### Comparison of Our Results with the Previous Findings

The QTLs detected in the present study, including 35 QTLs controlling fiber quality traits and 11 QTLs controlling yield traits, were mapped to 11 chromosomes (Chr2, Chr3, Chr5, Chr8, Chr10, Chr11, Chr12, Chr13, Chr14, Chr17, and Chr20). Among these QTLs, three (qLP-17-7, qLP-17-8, and qLP-3-12) were consistently detected in all three generations, and 13 were consistently detected in two generations (qFL-14-5, qFL-14-6, qFL-14-7, qFL-2-7, qFS-14-5, qFS-14-6, qFS-14-7, qFS-2-7, and qFU-20-9 detected in F_1_ and F_2_; qFM-17-7, qFM-17-8, qFM-3-12, and qLP-12-13 detected in F_2_ and F_2:3_) (S4 Table). Fifteen QTLs detected in the present study were reported in previous studies, including qFL-2-7, qFS-2-7, qBW-11-27, qBW-12-13, qLP-12-13, qFL-14-5, qFS-14-5, qFS-14-5, qFM-17-7, qFM-17-7, qFU-17-5, qLP-17-5, qFL-20-3, qFS-20-3, and qFS-20-15 [40–41; 48–50], based on the common markers harbored in the same confidence intervals of the same chromosome positions where these QTLs were mapped. Eight QTLs (qLP-17-8, qLP-3-12, qFL-14-6, qFL-14-7, qFS-14-6, qFS-14-7, qFM-17-8 and qFM-3-12) are reckoned to be novel stable QTLs. The detection of QTLs in multiple generations and different genetic backgrounds was suggestive of the stabilities of the genetic effects. The special attentions should be paid to these stable QTLs because the quantitative traits are usually susceptible to the various environmental factors, and the stable QTLs might increase reliability and efficiency of selection and play important roles in fiber yield and quality improvement via MAS [51–56].

### QTL Cluster and Linkage Distribution

Cluster distribution of QTLs is a relatively common phenomenon [14, 17, 53, 55–58]. Said et al. [59] comprehensively analyzed 2,134 previously reported QTLs in intra- and inter-species populations and detected numerous QTLs, which were distributed in clusters in certain chromosome regions in the specific populations. In the present study, QTLs controlling different traits were also detected at the same SSR marker locus. QTLs for FL and FS were mapped to the neighboring regions of markers HAU1980b on Chr2, NAU3648, NAU5421 and HAU1980a on Chr14, and CGR5565a, NAU5013, and NAU3665 on Chr20. QTLs for FU and LP were mapped to the neighboring regions of marker NAU5013 (S4 Table). QTLs for FM (qFM3-12, qFM-17-7, and qFM-17-8) were consistently detected in the neighboring regions of the molecular markers linked to the QTLs for LP (qLP-3-12, qLP-17-7, and qLP-17-8), suggesting that these major QTLs are closely linked to the same markers in the introgressed Hai1 segment, thereby providing an explanation for the significant correlations between the two traits in all three generations (Table 3). These results indicate that these loci might function as pleiotropic genes or are closely linked to the various other genes.

The chromosome segments of these QTL hotspot clusters could be useful for molecular breeding based on common molecular markers [59]. When the chromosome segments clustered both the favorable alleles of the QTLs for cotton fiber quality and yield traits, it could be more easily used for simultaneous improvement of traits. However, when the chromosome segments clustered the negatively correlated favorable alleles of QTLs, it would be very difficult to simultaneously improve these traits. An in-depth study of this linkage mechanism and breaking the linkage between cumbersome genes would play a significant role in cotton molecular breeding.

### Sources of QTL Synergistic Genes

Among the QTLs for fiber quality traits mapped in the present study, the synergistic genes for 14 QTLs were from CCRI36, whereas the synergistic genes for 21 QTLs were from Hai1. Among the QTLs controlling cotton yield traits, the synergistic genes for 8 QTLs were from CCRI36, whereas the synergistic genes for 3 QTLs were from Hai1. These results suggest that fiber quality or yield QTLs are not necessarily derived from the superior parent and the parent with relatively poor traits can also contribute genes that favor fiber yield and quality. Our findings also indicate that introgression between upland cotton and sea island cotton may broaden genetic variations as well as increase the potential of favorable gene rearrangements.

## Supporting Information

S1 Table Statistical analysis of Hai1 introgression segments in the parental lines. (XLSX)

S2 Table Statistical analysis of Hai1 introgressed segment length in the three generations. (XLSX)

S3 Table Statistical analysis of Hai1 substitution segments in the three generations. (XLSX)

S4 Table QTLs related to fiber quality and yield traits detected in the three generations. (XLSX)
